# Beyond America's War on Drugs: Developing Public Policy to Navigate the Prevailing Pharmacological Revolution

**DOI:** 10.3934/publichealth.2015.1.142

**Published:** 2015-03-30

**Authors:** Andrew Golub, Alex S. Bennett, Luther Elliott

**Affiliations:** National Development and Research Institutes, 47 Prospect Parkway, Burlington, VT 05401, USA

**Keywords:** drug war, history, social control, epidemics, performance enhancement, culture, postmodernism, prevention, controlled use

## Abstract

This paper places America's “war on drugs” in perspective in order to develop a new metaphor for control of drug misuse. A brief and focused history of America's experience with substance use and substance use policy over the past several hundred years provides background and a framework to compare the current Pharmacological Revolution with America's Nineteenth Century Industrial Revolution. The paper concludes with cautions about growing challenges and provides suggestions for navigating this revolution and reducing its negative impact on individuals and society.

## 1. Introduction

The “War on Drugs” is not an actual war. It is a metaphor. Metaphors can greatly help in understanding the nature of a problem and its likely resolution. Metaphors allow one to understand a complex problem in terms of a simpler one. The drugs-war metaphor says that the complex drug problem facing the nation can be understood as similar to an invasion by a foreign army like the British in 1812, the Japanese in 1941, and the attack on the World Trade Center in 2001. Once we accept the metaphor as valid, then we continue the metaphor by saying that the solution for the analogy is also the solution for our target problem. Thus, fighting the invader—drugs, drug dealers and drug users—provides a way to resolve the drug problem.

However, metaphors can be a dual-edged sword. Slavish obedience to a metaphor risks accepting an oversimplification of the problem which can place undue hope on a naive solution. We contend the drugs-war metaphor fails on many levels. On one level, we have trouble identifying the enemy. The drugs we worry about today, such as cocaine and heroin, were once accepted by medical practitioners as miracle drugs. Some of the drugs that are popular today, such as OxyContin and Vicodin, are providing great medical benefit to many but also leading to abuse for others. Will these be tomorrow's demon drugs? This paper examines America's extended War on Drugs as well as the larger challenge of drugs and their associated problems. We first trace the history of America's relationship to psychoactive drugs before offering a theory of subcultural evolution and drug use. We conclude by suggesting that America is undergoing a Pharmacological Revolution that in many respects is similar to the Industrial Revolution. This new metaphor allows us to illustrate a potentially more effective approach to developing US drug policy based on a socio-cultural perspective. This paper limits its focus to the US and does not address the varied and inter-connected aspects of a global war on drugs.

## 2. A brief history of America's drug policy and the war on drugs

America's complex history of drugs and drug policy has been heavily affected by technological advances, population movements, urbanization, and the restructuring of social and economic life. A central issue has been a massive decline in informal social control and an attendant rise in the role of the State with its formal mechanisms of socio-economic regulation. This paper starts with a description of the massive cultural changes during the Industrial Revolution. This serves multiple purposes: first, it lays out the historical context of our ongoing development of drug use and drug policy; second, it presents examples revealing how substance use is intimately tied to a social context that can undergo change when the context changes; and third, it provides insight into the massive cultural changes that took place during the Industrial Revolution which illustrates the magnitude of changes that could result from the current Pharmacological Revolution. We then describe other cultural and substance use changes occurring in the Progressive Era, around the time of World War II, around the Vietnam War and as part of the current Pharmacological Revolution in the Twenty-First Century. Table 1 summarizes the various historical periods and the drugs involved. After the historical review, the paper provides our recommendations for drug policy development based on viewing all of the current drug problems and possibilities. (not just use of those that are illegal) as based in a Pharmacological Revolution rather than a War on Drugs.

**Table 1.** Key periods in the history of America's experience of drug use.

Period	Approximate Yearsin America	Some key substances characterizing the period
Colonial Period	before 1800	alcohol
Early Industrial Revolution	early 1800s	coffee
Industrial Revolution	1800s	morphine, heroin, cocaine
Progressive Era	1890–1929	*Federal anti-drug legislation*Harrison Narcotics Act of 1914
Modern Period & World War II	1930–1959	amphetamines
Vietnam War &Youth Movement	1960–1979	heroin, marijuana, LSD, PCP
Pharmacological Revolution	1980–Present	alcohol, marijuana, heroin, crack, Valium, OxyContin, Prozac, Xanax, Ritalin, Adderall, Viagra, steroids

## 3. A Drug Eras Perspective

We organize our historical review as a series of drug eras. This framework provides a basis for studying drug use and drug policy within context and helps illustrate how the interplay between drug use and policy are heavily context dependant. Much research has documented that the popularity of different illegal drugs rose rapidly and then fell in the late Twentieth Century, constituting what appear to be distinct eras [1]–[6]. The lead author has examined various drug eras including the Heroin Injection Era of the 1960s and early 1970s [7], the Crack Cocaine Era of the mid-1980s to early 1990s [8] and the Marijuana/Blunts Era starting in the 1990s [9].

Drug eras in the late Twentieth Century tended to include four distinct phases: incubation, expansion, plateau, and decline [10],[11]. A drug era typically starts among a highly limited subpopulation participating in a specific social context constituting an incubation phase. For instance, the Heroin Injection Era grew out of the jazz music scene [12],[13]. The Crack Era started with inner-city drug dealers at after hours clubs [5]. The Marijuana/Blunts Era was based in the hip-hop movement [14].

During the expansion phase, the pioneering drug users or medical advocates successfully introduce the practice to the broader population. In a very broad review of the literature, Everett Rogers [15] identified that when ideas spread they tend to spread with increasing rapidity, whether it involves a new consumer product, fashion, teaching method, or agricultural technique. Mathematically, many aspects of these “diffusion of innovation” processes are analogous to disease epidemics. The primary difference between social diffusions and disease epidemics is what is being spread—an idea or behavior as opposed to a bacteria or virus. People have agency regarding whether they adopt a behavior, such as use of a new drug, and many people choose not to. Regarding drugs, individual susceptibility to use varies greatly according to social networks, social and economic status, societal and structural constraints, and personal identity. It is the rapid growth in popularity during the expansion phase that shocks law enforcement, the media and other social institutions leading them to use and abuse the term “drug epidemic” to arouse concern and serve political agendas [16]–[18]. In this paper, we use the less emotionally charged phrase “drug era” to emphasize the cultural aspects of the phenomenon.

Drug eras eventually reach a plateau phase when everyone most at risk of the new drug practice either has initiated use or at least had the opportunity to do so. For a time, widespread use prevails. Eventually, the use of a drug may go out of favor. This leads to a gradual decline phase of a drug era. This shift can be precipitated by emergence of drug-related problems, the availability of a more desirable or fashionable drug, a policy intervention aimed to curtail use, a general cultural shift or a combination of these factors. During this phase, new conduct norms emerge that hold that use of a drug is bad or old-fashioned. The subsequent new norms and policies then compete with the prevailing pro-use norms. During the decline phase, a decreasing proportion of youths coming of age become users. However, the overall use of the drug generally endures for many years as some users continue their habits. We now use this framework to examine earlier drug eras. What differentiates many of these earlier eras from those of the late 20^th^ century is that usage of the drugs of concern involved medical and social reasons as opposed to counter-cultural activity.

## 4. Early Industrial Revolution––alcohol and coffee––a focus on productivity

America's war against the consumption of mind-altering substances. (including alcohol) can be traced to Seventeenth Century England and its experience with early industrialization [19],[20]. During the Industrial Revolution, cities grew in both size and importance. Concomitantly, there was a decline in the importance of the extended family as a social and an economic unit. As a result, there was a decline in the informal social controls grounded in family, community, and church [21],[22]. Urban life was increasingly being viewed as disorderly. Government increasingly stepped in to fill the void with laws and mechanisms of enforcement.

During this period, major changes in social and economic structure required changes in substance use norms and practices. Prior to the Industrial Revolution, the British consumed alcohol widely and throughout the day. During Britain's Industrial Revolution, alcohol use came to be replaced by coffee which was more consistent with social expectations for being alert, punctual, rational and productive. An anonymous observer in 1674 clearly noted this shift as it occurred [19]:

Coffee-drinking hath caused a greater sobriety among the nations; for whereas formerly apprentices and clerks with others, used to take their mornings' drought in ale, beer or wine, which by the dizziness they cause in the brain, make many unfit for business, they use now to play the good-fellows in this wakeful and civil drink.

Industrialization and the growth of cities brought about significant changes in the organization of social and economic life. Family-based economies and communities that had been self-sufficient eroded as the young experienced a push away from an agrarian lifestyle and toward factory work. Unlike the rural family-based farm economy, at least in the eyes of supervisors, factory work required head work, discipline, and punctuality––all of which could be best achieved through sobriety. Caffeine as delivered in coffee was considered the antithesis of alcohol and beer; its stimulant effects produced good workers, the fuel of industrial capitalism. In this regard, coffee promoted hard work while beer was thought to produce lazy workers and slow the pace of economic growth. Coffee went hand in hand with the new rhythms of industrial work. Indeed, Brian Cowen [23] contended, “Coffee was the Protestant ethic in liquid form.”

New industry-driven labor requirements and expectations of acceptable behavior soon transferred over to the British colonies. As early as 1633, Massachusetts Bay Company Governor John Winthrop discontinued the practice of “drinking healths”. (a toast to one's health) in the colony and stipulated that a Governor's permit was necessary to sell liquor [24]. A century later, Benjamin Rush, who penned his name to the Declaration of Independence, published his now famous *An Inquiry into the Effects of Ardent Spirits upon the Human Body and Mind*. This work heralded the emergence of the early temperance movement conceptualizing chronic drunkenness as an “odious disease” and condemning the practice from both a medical and moral perspective [24],[25]. This shift between an early Alcohol Era into a Coffee Era was quite profound because both substances were extremely popular. Their use was widespread across subpopulations differing by class, race, ethnicity, and gender. Consumption of both substances persists today and for many is integrated into a contemporary lifestyle. Later drug eras tended to affect a more limited segment of the population.

## 5. Industrial Revolution––morphine, cocaine and heroin––the use of miracle drugs

The Industrial Revolution was also characterized by an increasing pace of technological innovation in all areas, including medicine. The history of those substances most often constructed as drugs of abuse date to the identification and synthesis of a series of miracle drugs that revolutionized medicine in the 1800s—morphine, cocaine and heroin [2],[26]–[29].

Morphine was first isolated, developed, and distributed in the early Nineteenth Century. By the mid-1800s, the use of morphine had become commonplace for the medical treatment of pain. The development of the hypodermic syringe in 1853 allowed more effective delivery and, as a side effect, provided users with an intense morphine high. Morphine as delivered via the hypodermic syringe was used widely during the Civil War to treat wounded soldiers [30]. Dessa Bergen-Cico [27] in her book *War and Drugs* noted how America's wars have often been associated with the spread of drug use practices. The medical needs of Civil War soldiers fostered the spread and rapid expansion of the use of morphine. Use continued after the War reaching a peak by 1880. At mid-century, the use of opium was also spreading in various tinctures provided by doctors and patent medicine purveyors primarily to a largely female and middle-class clientele for a wide range of ailments such as headaches or menstrual cramps [30]. By the 1880s there was a wave of addiction among Civil War veterans, middle class housewives, and doctors [30]. This Morphine Era took place over the course of more than an entire century and finally went into decline during the progressive era as these miracle drug came to be thought of as a public menace [31],[32]. Morphine is still widely used in medicine today but is no longer a substance of widespread abuse. Like coffee, the positive use of this substance appears to be well integrated into our contemporary lives.

Two other new miracle drugs followed morphine's trajectory leading to a Heroin Era and a Cocaine Era at the end of the Nineteenth Century [30],[33]. Cocaine enjoyed a variety of miracle medicine and patent medicine uses. It was also used for performance enhancement and outright recreational use. Its use as a stimulant in such drinks as Coca Cola which at the time combined cocaine, caffeine and sugar was widely popular too as a boost to help workers perform the routinized tasks associated with industrial labor or as an effective tonic. Cocaine was removed from Coca Cola in 1903. Heroin was first marketed by Bayer as a powerful cough suppressant and pain reliever in the 1890s. A cough suppressant was a highly desirable and perhaps “heroic” drug at a time when many people were dying of tuberculosis and the cause of this coughing-related disease was still unknown.

## 6. Progressive Era––a decline phase––institutionalizing formal social control

America's War on Drugs and emphasis on supply reduction took shape during America's Progressive Period at the turn of the Twentieth Century. Around this time, people were developing problematic habits involving morphine, opium, cocaine, and heroin. Additionally, use of these drugs had expanded to new populations including members of the lower classes and ethnic/racial minorities, which contributed to the declining image of these drugs. Users became stigmatized as either hedonists or criminals and the drugs themselves became demonized [20],[34],[35]. These problems and perceptions led to the eventual end of the early Morphine, Cocaine and Heroin Eras through the formal social controls of regulation and enforcement.

Drug abuse control was one of many state-sponsored social engineering programs of the Progressive Period bringing economic and social regulation designed to provide urban infrastructure, social welfare institutions, education, and to enhance productivity. For many, life became more complex in urban environments as new needs arose. The unprecedented growth of many cities outpaced municipal governments' abilities to adequately meet the demand for services. In this pivotal period, city planners and residents increasingly associated urban problems with recent immigrants and migrants as populations of cities became more diverse and less white.

In Progressive America, newly minted experts and professionals engaged the myriad problems thought to be associated with drugs and their use. Medical experts vied with law enforcement to control drug users. In the 1880s, for example, when morphine, cocaine and heroin were considered medicines, users were considered patients. By the early 1900s, doctors were less inclined to prescribe opiates to patients due to the growing acceptance of the “germ theory” of disease and the increased use of non-addicting analgesics such as aspirin for pain [36]. The use of the opiate-based semi-synthetic heroin as well as cocaine for recreation, however, remained widespread and use became largely concentrated in stigmatized populations, especially poorer, urban males [30],[36]. Newspapers began drawing the public's attention to the drug problem with a barrage of drug-war language, such as a 1900 *New York Times* story which declared a “War on Opium” [37].

Federal drug regulation started with the 1906 Pure Food and Drug Act requiring that products containing drugs be labeled with content and dosage. This reduced the widespread promotion of medicines containing strong substances and their inadvertent use. In 1914, the Harrison Narcotics Act effectively criminalized non-medical use of cocaine and opiates. Subsequently, domestic drug convictions and international drug control efforts intensified. During this period the fight against drugs was waged by both the new law enforcement and medical experts both seeking to establish their ownership of the drug problem. A split trajectory emerged by 1930 whereby better-to-do medically prescribed users were treated as patients, in hospitals. Poorer, recreational street users were treated in the criminal justice system [31],[35],[38],[39].

Moving into the Twentieth Century, the law enforcement model with its stigmatized user population became the focus of the War on Drugs. By 1930 the iconic image of the drug user was no longer a Civil War veteran or a housewife but a lower income, minority laborer. Moreover, drug use for non-medical purposes became firmly associated with self-indulgent behavior, which many contemporaries viewed to be characteristic of a larger decline in traditional morality [30],[35]. These shifts illustrate how expanded migration, racism, and class issues colored the escalating War on Drugs.

## 7. World War II––amphetamine––enhancing productivity again

World War II was instrumental in shaping drug use patterns over the next decades. By the 1930s, a new class of long-acting compounds, amphetamines, increasingly made inroads into American society. Amphetamines were often prescribed by doctors to counter depression and blunt the sense of fatigue. Pep pills containing amphetamines were also widely distributed to soldiers during World War II and the Korean War [27]. Many soldiers became dependant and continued use after the war. Though prescription drug classes existed as early as 1936 and stricter regulation accompanied the Durham-Humphrey Amendments of 1951, many of these prescription drugs were not strictly regulated until the 1965 Drug Abuse Control Amendments. Thus, many veterans continued using the drugs after the wars easily obtaining their supplies. Amphetamines were sold illegally at truck stops primarily to help truck drivers stay alert but also facilitating the expansion of use across the country, flooding cities from coast to coast [40],[41]. An article in the *New York Times* [42] described how a federal crackdown under way was now targeting the stigmatized pep pills as follows, “Amphetamines, potent stimulants sold commercially as Benzedrine and Dexedrine, have emerged in recent years as a major cause of delinquency…. Excessive use of the drugs causes a breakdown of social and moral barriers.”

Undeniably, the 1950s marked a period of intense effort by state and Federal government forces to eliminate the foreign enemy and the enemy within [2],[38]. New efforts to control American borders and limit the illegal importation of drugs were initiated alongside a series of escalating penalties for drug use, possession, and sales. The 1951 Boggs Act and 1956 Narcotics Control Act carried newly created mandatory minimum penalties for narcotics violations. The fervor for the War on Drugs was advanced as this effort was conflated with America's Cold War against communist expansion. The drug using enemies within were considered especially susceptible to communist propaganda and therefore at risk of becoming spies [32],[43]. Accordingly, advocating for anything other than a full blown War on Drugs called in question a politician's patriotism. In 1954, California Governor Goodwin J. Knight's told the Conference on Youth and Narcotics that, “Dope peddlers…deserve no mercy whatsoever. Remember that they represent a greater and more deadly evil than a man with a loaded gun pointed right at your heart.” [38].

## 8. Vietnam War––heroin––drug, set and setting

The 1960s and early 1970s were a period of substantial domestic turmoil. Social movements focused on ending racism, concentrated poverty, and the war in Vietnam. American cities were under siege from protests that turned violent. Youths and young adults heralded a period of widespread experimentation with a broad variety of drugs. (many of which were relatively new) including LSD, PCP, barbiturates, amphetamines, heroin and marijuana. In 1971, President Richard Nixon officially responded by declaring a “War on Drugs.” [2],[44],[45]. Nixon's War on Drugs was not a unique new approach nor was he addressing a new problem. Rather, Nixon's War on Drugs represented a simplistic continuation of law enforcement policies toward an ongoing problem. However, the problem was getting more complex, the number of drugs available was increasing, and our understanding of the drug use experience was being challenged by new theoretical insights.

These insights grew out of studies of drug use by soldiers during the Vietnam War [27]. There was widespread concern that the return of soldiers addicted to heroin from Vietnam would continue their use [11],[27],[30]. Systematic research at this time however strongly suggested otherwise. In 1971, Lee Robins conducted a survey of service members in Vietnam and veterans who had returned to civilian life [46],[47]. She found that close to half. (45%) of veterans had used opium or heroin while in Vietnam and 20% said they had been addicted and reported typical withdrawal symptoms. Remarkably, only 5% of the men who became addicted in Vietnam relapsed within 10 months after return home to civilian life, and only 12% relapsed even briefly within three years. Her findings highlighted the importance of context as a formidable factor shaping drug-using experiences. Robins concluded that despite popular rhetoric,

Soldiers in Vietnam had no special vulnerability to narcotics. They used heroin because it was inexpensive, unadulterated, and easily available, alternatives were few, disapproving friends and relatives were far away, and they felt that their war service was somehow not part of their real lives. When their situation changed, most of them had no difficulty giving up heroin, and that should not have been surprising.

Norman Zinberg [48] built upon Robins' insights into the importance of context for drug-using practices. He described how factors operating within three nested domains—drug, set, and setting—affected the substance use experiences. He explained that the action of a “drug” describes the properties that affect an individual's body. Today we understand that much of this effect is manifest across the dopamine pathway. “Set” is a user's psychological expectations or mindset surrounding the consumption of a drug that further influences the experience. A user's set is influenced by their personality and internal states of mind and brings into consideration such elements as depression, happiness, stress, and anxiety. “Setting” includes the environmental, social, and cultural context in which substance use takes place. The substances available and the significance society and the individual come to attach to the substances influence a person's experience or relationship with a substance. In this manner, drug experiences are context dependent. Zinberg suggested that the context or setting is much more than a collection of distal antecedents. It is an organic system with its own internal logic based in a worldview. In this manner, Zinberg challenged the War on Drugs' focus on drugs and drug users rather than contexts that might engender problematic usage patterns [44],[48]. This framework consolidated important insight into drug use and still has a strong influence on research and policy [11]. The perspective stresses that moderate and controlled use of drugs is achievable, more common than previously thought or acknowledged, and most centrally context dependant. This directly challenges older stereotyped notions that any drug use would result in crippling addiction, espionage, crime, and the decline of America—oversimplifications that supported a War on Drugs.

## 9. The Twenty-First Century––a pharmacological revolution

Historically, we have seen a variety of reasons for taking drugs. Today there are many more drugs, more people using drugs, and numerous reasons to use them. We suggest that it is not the drugs themselves that we need to control, rather it is the misuse of these drugs that is problematic. We further suggest that social policy interventions would be more effective if they took this more expansive view. Reducing misuse involves understanding the reasons people use drugs, their mindset, and the context surrounding use. This provides insight into the underlying basis of our nation's drug problems. The following list catalogues a range of very different reasons for drug use varying from the most personally indulgent and individualistic to the most integrated into mainstream culture, although potentially misguided:

- Recreation/Enjoyment
- Making Meaning
- Medication/Self-Medication
- Cosmetic Pharmacology
- Performance Enhancement

Typically when we think about illegal drug use we think about people taking drugs to get high as a leisure activity for recreation or enjoyment. However, use of drugs can involve much more than seeking an altered state of conscience. There can be a major element of social identification involved. Drug use can represent a larger affiliation with a group or an idea. Social activities, use by friends, popular images, references in music, myths, availability, potential legal consequences, and youthful rebellion can impart a greater significance to the behavior. In this manner, drug use occurs within a cultural context and is part of the process by which people construct meaning in their lives on their postmodern journeys. Based on analysis of the succession of drug eras in the late 1900s, the lead author developed a theory of subcultural evolution and drug use as a partial explanation of the socio-cultural forces involved [10].

**A theory of subcultural evolution and drug use**: Drug use emerges from a dialectic of the prevailing culture. (and especially drug subcultures) with individual identity development. Use of a drug is clearly an individual's decision but it is the prevailing drug subcultures and each person's place relative to them that impart a greater significance to the activity. Conversely, individual decisions to adopt, adapt or reject aspects of the prevailing drug subcultures cause the subcultures to evolve as well as lead to the emergence of new ones.

Defining culture and subculture is complex and sometimes controversial [49]. However, understanding the relationship between culture and drugs is essential to the cultural approach to drug abuse control that we seek to promote in this paper. Culture has taken on different meanings for different groups over time. In 1871, Sir Edward Burnett Tylor, a leading anthropologist, provided a concise statement of the monumental and comprehensive nature to culture that now represents a classical formulation [50]:

Culture or civilization, taken in its wide ethnographic sense, is that complex whole which includes knowledge, belief, art, morals, law, custom, and any other capabilities and habits acquired by man as a member of society.

The classical perspective identifies a larger gestalt to social context that compels individuals to engage in various behaviors and attach significance to them. On the negative side, this older viewpoint clearly downplays diversity within a society and the potential of personal autonomy. A postmodern sensibility emphasizes the multiplicity of prevailing cultural frameworks, the interacting of themes, and the centrality of individual agency [51],[52]. Ulrich Beck [53] described a “reflexive cosmopolitization” whereby individuals build their identities based on multiple affiliations leading to a broad intermingling of ideas and behaviors without reference to national borders. Ann Swidler [54] provided a pragmatic view of culture as a toolkit of habits, skills and styles from which actors construct their strategy of actions and create meaning in their lives. Dick Hebdige [55] noted that subcultural identity manifests in decisions about self-presentation such as clothing, style, language, and use of public space.

There is substantial evidence that for many who become heavily involved with a drug era that their drug use is very much about identity and less about dropping out of society to pursue a leisure activity. Not every user becomes heavily involved with a drug era or the primary drug that comes to define that era. However, focusing on heavy users within an era provides insights into the context in which use becomes problematic and ultimately a window into prevailing culture. Ed Preble documented how many users during the Heroin Injection Era came to organize their daily lives around their habit: performing various hustles, nondrug crimes, a variety of drug sales/distribution roles, chasing the best bag of heroin, locating a safe place to inject, persuading others to share drugs or needles, avoiding police, and finding free food, shelter, and clothing. Drug users often described their heroin habit and associated activities as “Taking care of business,” an activity that provided them with a sense of purpose that for many born into poverty could not have been achieved in conventional society [56]. Similarly, during the Crack Era users attached symbolic importance to their extended efforts to obtain money and drugs during binges of use lasting for hours and even days. They referred to their efforts as missions adopting jargon from Star Trek [57]. Our larger point is that dealing with problems of drug abuse involves more than presenting users and potential users with a cost-benefit calculation of whether they should enjoy the benefits of a leisure activity or not. It is necessary to consider the complex and personal process by which individuals find meaning in life and how.

These first two reasons for drug use—enjoyment and making meaning—represent reactions contrary to prescribed mainstream norms for drug use and are mostly associated with illegal drugs. Other uses for drugs represent efforts to cope with contemporary life, not necessarily escape, and mostly involve drugs that are currently legal with a prescription. Self-medication can be understood as an effort to keep problems in check in order to otherwise participate in mainstream society. Individuals may also self medicate to deal with disorders or pain when they lack the resources to obtain mainstream services. In a sense, this represents a neutral use of drugs—to be normal or be able to operate in light of basic mainstream expectations. However, the use of drugs has raised the question, “What is normal?” Given that improved functioning can be achieved with drugs, it has raised the additional question, “Why settle for normal, when one can do better?”

Indeed, medical and pharmacological practice has clearly been at the forefront of this change. Joe Dumit [58] argued that there has been a fundamental philosophical shift over the past several decades. In the Nineteenth Century, medicine was understood as a cure, often a one-time administration, that returned the body to its normal, otherwise healthy status. Dumit noted a new pharmaceutical worldview that has accelerated since the 1990s that presumes that the body is “inherently ill” requiring maintenance medications. We now have various drugs for treating attention deficit hyperactivity disorder. (ADHD) such as Ritalin and Adderall [59]. We also have a variety of drugs for controlling depression and anxiety such as Valium, Prozac, and Xanax [60]. And a proliferation of drugs to improve sexual performance impeded by erectile dysfunction such as Viagra [61]. Treatments have been discovered for conditions and concerns with which people had to learn to cope. This potential has also raised concern that there may be over diagnosis of problems by care providers and drug manufacturers in a cynical pursuit of profits. Direct-to-consumer marketing by pharmaceutical companies adjures viewers to check with their doctor or pharmacist as to whether a new drug may relieve their condition or improve their performance [62],[63]. The implication is that problems or concerns that one may face are treatable through drugs leading to what Peter Conrad referred to as the “medicalization of society.” Overall, there has been a massive increase in substance use, much of which may be unnecessary. There has been an increase in the number and quantity of drugs that can be potentially diverted. There has also been a growing concern with the misuse of drugs by the person for whom they were prescribed including such aberrant behaviors as complaining about the need for more drugs, unsanctioned dose escalation, concurrent use of alcohol, or alternative route of administration such as sniffing or injecting drugs originally intended for oral use [64]. In this way, doctors are losing control over the use of those drugs that are under the prescription system.

For some, preference is starting to replace need as a basis for drug use. Peter Kramer's [60] influential book, *Listening to Prozac*, raised serious questions about how we decide what are normal feelings for people to experience, what personality characteristics should be considered problematic, and who decides. Kramer reported a variety of curious responses to Prozac by patients such as: “I felt more like myself when I was on the drug than when I was not,” “It was a mood brightener,” and, “My friends liked me better when I was on drugs.” These observations illustrate “cosmetic pharmacology,” the use of drugs to enhance your appearance just as one might have cosmetic surgery to remove fat, reduce frown lines or enhance one's breasts.

For others, drugs have become a way to enhance their performance in order to keep up with the demands of contemporary life. This is especially the case with amphetamines. The question arises as to the extent that Adderall and other stimulants are being used for performance enhancement either with medical supervision, as an aberrant behavior outside of prescribed use, or through diverted supplies [59]. In their book *Game of Shadows: Barry Bonds, BALCO, and the Steroids Scandal that Rocked Professional Sports*, Mark Fainaru-Wadu and Lance Williams [65] discussed this larger problem with regard to baseball, running and other professional and Olympic sports but especially with regard to Barry Bonds' stellar career and the network developed to help him reach his maximum potential by using steroids and other performance enhancing drugs. This raises the concern that once a few athletes take performance enhancing drugs, others can choose not to, but only at the risk of forsaking their career goals [66]. This represents a form of social coercion urging individuals to use drugs to enhance their performance.

Performance enhancing substance use has been common in the military, especially during conflicts, and not just for recreational purposes [27],[67]. In the Twenty-First Century, the U.S. Military has been engaged in two extended conflicts in Afghanistan and Iraq, referred to as Operation Enduring Freedom. (OEF) and Operation Iraqi Freedom. (OIF). Soldiers routinely take substances such as Dexedrine, NoDoz and Red Bull commonly called “go pills.” As a come down to obtain needed sleep and to suppress anxiety, soldiers routinely take other substances including Ambien and Restoril, commonly called “no-go” pills. To deal with pain while deployed and after returning, many soldiers are taking powerful new opioids including OxyContin and Vicodin. Because of the widespread use of drugs by military personnel and veterans, a *New York Magazine* article dubbed this, “The Prozac, Paxil, Zoloft, Wellbutrin, Celexa, Effexor, Valium, Klonopin, Ativan, Restoril, Xanax, Adderall, Ritalin, Haldol, Resperdal, Seroquel, Ambien, Lunesta, Elavil, Trazodone War” [68]. Eventually, OEF/OIF veterans need to integrate into the rhythms of civilian life which are generally less intense than combat experiences. This involves possibly reducing the use of go and no-go pills. Many OEF/OIF veterans are also dealing with ongoing use and dependence on opioids.

## 10. Toward a cultural perspective on drug policy

The Federal administration signaled its interest in moving past the drugs-war metaphor. Gil Kerlikowske, former Director of the Office of National Drug Control Policy or Drug Czar, commented that “Regardless of how you try to explain to people it's a ‘war on drugs’ or a ‘war on a product,’ people see a war as a war on them. We're not at war with people in this country.” [69]. Consistent with this promise, the 2011 *National Drug Control Strategy* contains no mention of a War on Drugs and instead focuses upon drug abuse as a public health concern [70]. This goes back a hundred years returning to the competition between law enforcement and medicine for ownership of the drug problem. However, this viewpoint still misses the larger context in which drug-related problems are generally based. From Zinberg's perspective this is still a focus on the drug and the set. We contend that the greatest advances in resolving our drug-related problems can be obtained by focusing on setting and in particular on how setting is transforming over time.

Our approach to alleviating drug misuse and its associated problems starts with recognizing drugs for what they are. They are technologies! In this regard, our drug problems are similar to problems we face with other new technologies such as cell phones, the internet, microwave ovens, plastic, cars, refrigeration, and nuclear energy. These technologies bring advantages. However, in the process they have changed our world forever, just as the growth of factories reorganized our lives during the Industrial Revolution. We cannot go backwards! Traditional societies like the Amish avoid the problems of new technologies by completely shunning their use. However, in the larger competitive society this is not practical. We cannot declare war on cell phones and eliminate their use. Similarly, a war on the internet appears undesirable and likely futile.

Continuing our analogy, we formally distinguish our current postmodern period as a Pharmacological Revolution. We contend this provides us with a more accurate metaphor for policy development than sustaining a War on Drugs. Based on our analogy, we make three major predictions:

1. The world will be qualitatively different at the end of the revolution in ways that we could not understand and would not have accepted before the start of the transformation.

This is a very humbling thought. Moreover, there is no simple and obvious path for all to navigate this revolution leading to the next predictions:

1. There will be pain and hardship during the transition as early adopters struggle with the collateral consequences of using new technologies.
2. There will be pain and hardship suffered by late adopters and non-adopters as the world around them changes and leaves them behind.

Accordingly, we contend that there needs to be a fundamental shift in how drug policy is developed, a change in metaphor. This new perspective would seek to help individuals find their way through the prevailing Pharmacological Revolution. Towards this end, we make several explicit recommendations:

1. Study how drug use technologies affect society. Not just from a pathological perspective, but also consider the potential of controlled use, the impact of one's use on others around that person, and the larger impact on society. This would involve increasing research by social scientists into controlled substance use that solves problems and enhances individual lives. Current research funded through the National Institute on Drug Abuse tends to examine pathological concerns and emphasizes biological concerns over cultural and social developments.
2. View regulations as provisional as drug use and associated consequences play out differently and can vary over time and across locations. Hard and fast regulation and enforcement without an understanding of context impedes orderly change to society. As in the past, our base of knowledge and experience at this time limits our ability to make the best permanent decisions. Accordingly, we need a range of regulatory instruments and monitoring procedures. As we move through this Pharmacological Revolution, policies can be tightened, loosened or otherwise revised with increasing information and experience.
3. Focus on education that will help individuals make good decisions that lead to healthy, productive, and fulfilling lifestyles. This stands in strong contrast to current drug education programs, public service announcements, and treatment programs that emphasize abstinence only. The next section discusses possible prevention programs further.
4. Provide culturally sensitive outreach programs tailored to those in need. We need to engage drug-users and the communities they are a part of to understand the nature of the problems they face. This will allow us to best inform and craft interventions that meet the often wide ranging drug-user and community needs, including the provision of treatment, risk reduction measures, and where appropriate, forms of punishment.

## 11. A cultural view on alcohol abuse prevention

Some of the most suggestive information about the cultural element to our substance abuse problems comes from cross-cultural studies of alcohol. There has been extensive research on the interrelationship between alcohol use, alcohol policy, and cultural norms. This work may provide potential insights for developing responses to the use of other substances bearing in mind their broader impact on society. Alcohol has been widely used over time and across societies. Moreover, there has been substantial variation in cultural relationships to alcohol. David Hanson's texts have explored alcohol history, literature and policy [71],[72]: *Preventing Alcohol Abuse: Alcohol, Culture and Control* and *Alcohol Education: What We Must Do*. Hanson pointed out that beverage alcohol has been used for enjoyment as well as medicinal, nutritional, antiseptic, analgesic and religious/spiritual purposes for millennia. But its misuse can be problematic. This has been long understood. Biblical writings point to both the value and danger of alcohol use [73]. St. Paul considered wine to be a creation of God and therefore inherently good. (1 Timothy 4:4), but condemned drunkenness. (1 Corinthians 3:16–17) and recommended abstinence for those who could not control their drinking. The puritan minister Increase Mather stated, “Drink is in itself a good creature of God and to be received with thankfulness, but the abuse of drink is from Satan; the wine is from God, but the Drunkard is from the Devil.” [quoted in 71].

This line of research holds that the challenge since early times has been to enjoy the benefits of alcohol while controlling the potential problems. This more nuanced concern differs dramatically from the drugs-war metaphor and further suggests that the efforts to prevent substance abuse should focus on cultural issues. Mandelbaum [74], a cultural anthropologist, argued that culture profoundly affects the interpretation of altered states brought as well as the actual physical response itself:

…The behavioral consequences of drinking alcohol depends as much on a people's ideas of what alcohol does to a person as they do on the physiological processes that take place. When a man lifts a cup, it is not only the kind of drink that is in it, the amount he is likely to take, and the circumstances under which he will do the drinking that are specified in advance for him, but also whether the contents of the cup will cheer or stupefy, whether they will induce affection or aggression, guilt or unalloyed pleasure. These and many other cultural definitions attach to the drink even before it touches the lips.

This idea, is consistent with Zinberg's focus on setting. Hanson offered several national case studies that ground experiences of alcohol use and abuse in distinct cultural contexts [71]. He noted that the Irish have high rates of alcohol abuse whereas Italians do not. He attributed Irish alcohol abuse to the traditional separation of males and females and that Irish males are encouraged to sublimate their sexuality and any emotional problems by “drinking it off with the boys.” In contrast, Italian alcohol use is traditionally integrated into family life where men and women and children drink moderately together, especially at meals. A recent World Health Organization report indicated that cross-cultural differences in alcohol use and abuse patterns persist to this day [75]. In Ireland as of 2005, 26% of the population had abstained from use of alcohol in the past 12 months. Among males, 43% had engaged in heavy episodic drinking in the past 7 days. Beer was the most common alcoholic drink consumed. (53%). In Italy, wine. (73%) was the alcoholic drink of choice. There were fewer abstainers. (18%) than in Ireland, yet a much lower rate of heavy episodic use among males. (11%). In both countries females were much less likely to engage in heavy episodic use; even still, the rate in Ireland. (14%) was higher than the rate in Italy. (8%). These findings are consistent with the possibility that the cultural differences in drinking between the Irish and Italians that Hanson spoke of may have persisted into the 21^st^ Century.

Regardless of the localized historical trends in substance abuse he reviewed, Hanson's research continues to have broader implications for the control of substance abuse today. It holds out the possibility that changing the context or influencing individuals' expectations and conceptualizations of use can control the emergence of heavy use and associated problems. Based on his cross-cultural and historical synthesis, Hanson [71] concluded that the following cultural factors are associated with lower rates of drinking problems:

1. Drinking is prescribed by social norms, not prohibited
2. Drinking is incorporated within social customs or religious observance
3. “Proper” drinking behavior is learned at an early age and within the home
4. Drinking accompanies meals
5. Drinking behavior is regulated by social norms for controlled use

Accordingly, Hanson [72] argued for alcohol policy and education that follow a sociocultural approach to foster controlled and responsible alcohol use and the avoidance of abuse.

## 12. Cultural approaches to reducing substance abuse

There is clear evidence of the advantage of taking a cultural perspective with regard to alcohol. We contend that drug abuse control programs incorporating a cultural perspective are also needed during this Pharmacological Revolution to identify and address the evolving contexts of legal and illegal drug use, misuse, and dependence. A key insight is that drug-related problems are not limited to illegal drugs. Use of drugs that are currently legal can result in problems, even when used with a prescription. Misuse of heroin, methamphetamine, marijuana, steroids, opioids and other drugs for recreation, self-medication, and performance enhancement represent topics of social concern worthy of further analysis. Unfortunately, our drugs-war metaphor has limited our analysis primarily to pathological use and toxicity of illegal drugs. An understanding that our nation is undergoing a Pharmacological Revolution suggests major changes in which drugs should be studied and how. In particular, it should be recognized that the use of any new drug, like a new technology, has the potential for broad changes on our society. Accordingly, we offer the following recommendation regarding ongoing research:

1. Research should analyze the ongoing social and cultural impact of new drugs. Currently, drug testing is mostly limited to the analysis of the efficacy and toxicity of drugs that are to become legal that are reviewed by the Food and Drug Administration before a drug is introduced into the nation's pharmacopoeia. Additional funding should be available to social and cultural researchers. (such as anthropologists, sociologists, and historians) for studies into the cultural impact of new drugs. This research should seek to establish how a person's use of a drug changes their life, the lives of others around them, and the impact for society as more people become users.
2. Research should analyze controlled use. The focus of most drug abuse research has been on various drugs' toxicity and ways to prevent access and use. However, toxic events can be avoided through controlled behavior. Research funding should be available to study the controlled use of drugs over time and in context. This research should involve drugs that are both currently legal and illegal bearing in mind that these distinctions can change over time. This research should also consider interactions with other common substances such as alcohol. For many people, it is unrealistic to presume that if they are taking a drug long-term such as opioids that they will abstain from any alcohol use. This research will provide essential health information regarding which drugs that are currently illegal or limited to prescription use might be made more broadly available and those that are currently legal that might be subjected to further restriction.
3. Research should analyze controlled use. The focus of most drug abuse research has been on various drugs' toxicity and ways to prevent access and use. However, toxic events can be avoided through controlled behavior. Research funding should be available to study the controlled use of drugs over time and in context. This research should involve drugs that are both currently legal and illegal bearing in mind that these distinctions can change over time. This research should also consider interactions with other common substances such as alcohol. For many people, it is unrealistic to presume that if they are taking a drug long-term such as opioids that they will abstain from any alcohol use. This research will provide essential health information regarding which drugs that are currently illegal or limited to prescription use might be made more broadly available and those that are currently legal that might be subjected to further restriction.

As a society we need to come to terms with our chemical and human potential to help individuals' construct healthy, productive and meaningful lives during this Pharmacological Revolution.
